# Biomarkers of immunotherapy in urothelial and renal cell carcinoma: PD-L1, tumor mutational burden, and beyond

**DOI:** 10.1186/s40425-018-0314-1

**Published:** 2018-01-25

**Authors:** Jason Zhu, Andrew J. Armstrong, Terence W. Friedlander, Won Kim, Sumanta K. Pal, Daniel J. George, Tian Zhang

**Affiliations:** 1 Duke University Health System, Durham, NC USA; 2 Duke Cancer Institute, DUMC 103861, Durham, NC 27710 USA; 3 University of California San Francisco, San Francisco, CA USA; 4 City of Hope Comprehensive Cancer Center, Los Angeles, CA USA

**Keywords:** PD-L1, Biomarkers, Immune checkpoint inhibition, Urothelial carcinoma, Renal cell carcinoma

## Abstract

Immune checkpoint inhibitors targeting the PD-1 pathway have greatly changed clinical management of metastatic urothelial carcinoma and metastatic renal cell carcinoma. However, response rates are low, and biomarkers are needed to predict for treatment response. Immunohistochemical quantification of PD-L1 was developed as a promising biomarker in early clinical trials, but many shortcomings of the four different assays (different antibodies, disparate cellular populations, and different thresholds of positivity) have limited its clinical utility. Further limitations include the use of archival specimens to measure this dynamic biomarker. Indeed, until PD-L1 testing is standardized and can consistently predict treatment outcome, the currently available PD-L1 assays are not clinically useful in urothelial and renal cell carcinoma. Other more promising biomarkers include tumor mutational burden, profiles of tumor infiltrating lymphocytes, molecular subtypes, and PD-L2. Potentially, a composite biomarker may be best but will need prospective testing to validate such a biomarker.

## Background

Over the past few years, immune checkpoint inhibitors (CPIs) blocking the programmed cell death receptor 1 (PD-1) pathway have emerged as treatments capable of improving the overall survival of patients with metastatic renal cell carcinoma (mRCC) and metastatic urothelial carcinoma (mUC) [1, 2]. Based on promising phase II and III clinical trials, the United States Food and Drug Administration (FDA) has approved nivolumab for renal cell carcinoma [2] and five PD-1/PD-L1 inhibitors (atezolizumab, pembrolizumab, nivolumab, durvalumab, and avelumab) for urothelial cancer [2, 3]. Due to relatively modest objective response rates (approximately 25% in mRCC and 15–21% in mUC), it is important to identify biomarkers predictive of response or resistance to immunotherapy [1, 3–5]. An ideal, clinically useful, predictive biomarker would dichotomize patients as potential responders or non-responders based on a positive or negative result. Moreover, an ideal biomarker assay would be consistent in its performance and threshold for defining a positive result. During clinical development of these CPIs, tissue-based assays for PD-L1 have been developed in parallel as a potential companion biomarker for CPI response.

PD-L1 is one of several ligands for the PD-1 receptor, and early trials in advanced mUC suggested that immunohistochemistry (IHC) staining for PD-L1 on paraffin-embedded tumor specimens would serve as a predictive biomarker [3, 6]. Based on this data, the FDA approved a companion PD-L1 assay (Ventana) in parallel with the approval of atezolizumab for mUC. However, with other clinical trials maturing, IHC staining for PD-L1 status has not fulfilled its promise as a predictive biomarker. Indeed, each PD-1/PD-L1 targeting therapeutic has been developed with its own PD-L1 companion diagnostic antibody, thereby limiting the clinical interpretation of PD-L1 status. In this review, we examine the characteristics of IHC staining for PD-L1 as a biomarker in both mUC and mRCC, as well as alternative predictive biomarkers which are currently in development. For convenience of comparison, we use objective response rates (ORR) defined as the rate of partial and complete responses, to discuss the treatment response across trials.

## Urothelial carcinoma and PD-L1 testing

The phase I study of atezolizumab in mUC was initially designed to include PD-L1-positive enriched cohorts, with a dose expansion cohort for all mUC patients regardless of PD-L1 status [6]. The Ventana IHC assay using the SP142 antibody (Ventana, AZ, USA) was used for PD-L1 assessment. Here, PD-L1 positivity was defined as IHC 2+ (≥5%) or 3+ (≥10%) PD-L1 expression on tumor cells or tumor infiltrating immune cells (including macrophages, dendritic cells, and lymphocytes). Forty-three percent (13/30) of patients with a positive PD-L1 tumor had an objective response to atezolizumab compared with only 11% (4/35) of patients with negative PD-L1 status, suggesting that PD-L1 IHC status might predict treatment response (Table 1). Following these results, several studies were conducted to confirm the anti-tumor activity of PD-L1 and PD-1 inhibitors in two distinct populations: patients with mUC who had progressed after platinum-based therapies and patients with mUC who were not candidates for first-line platinum-based therapies.

**Table 1:** Summary of PD-L1 assays and response rates in immune checkpoint inhibitor trials in metastatic urothelial carcinoma

Ref	Population	Drug	Target	Antibody for PD-L1 IHC Assay	Definition of PD-L1 positivity	ORR (PD-L1+)	ORR (PD-L1-)	ORR All Patients
[6]	Post Platinum	Atezolizumab	PD-L1	Rabbit SP142 (Ventana)	IHC 2/3 IC^a^	43% (13/30)	11% (4/35)	26% (17/65)
[7]	Post Platinum	Atezolizumab	PD-L1	Rabbit SP142 (Ventana)	IHC 2/3 IC^a^	23% (26/113)	10% (36/349)	13% (63/462)
[3]	Post Platinum	Atezolizumab	PD-L1	Rabbit SP142 (Ventana)	IHC 2/3 IC^a^	26% (26/100)	9% (19/210)	15% (45/310)
[1]	Post Platinum	Pembrolizumab	PD-1	Mouse 22C3 (Dako)	CPS ≥ 10%^b^	22% (16/74)	22% (41/186)	21% (57/270)
[5]	Post Platinum	Nivolumab	PD-1	Rabbit 28–8 (Dako)	PD-L1 ≥ 5% (TC)	28% (23/81)	16% (29/184)	20% (52/265)
[7]	Post Platinum	Durvalumab	PD-L1	Rabbit SP263 (Ventana)	≥25% TC or ≥25% IC	28% (27/98)	5% (4/79)	18% (34/191)
[10]	Post Platinum	Avelumab	PD-L1	Rabbit 73–10 (Dako)	≥5% TC	54% (7/13)	4% (1/24)	21% (8/37)
[50]	Platinum Ineligible	Pembrolizumab	PD-1	Mouse 22C3 (Dako)	CPS ≥ 10%^b^	51% (41/80)	23% (42/185)	31% (83/265)
[12]	Platinum Ineligible	Atezolizumab	PD-L1	Rabbit SP142 (Ventana)	IHC 2/3 IC^a^	28% (9/32)	20% (18/87)	22% (27/119)

*TC* tumor cells, *Ref* reference, *IC* percentage of PD-L1 positive immune cells in the tumor microenvironment, *ORR* overall response rate

^a^IHC 2 is ≥5%, IHC 3 is ≥10%

^b^Combined Positive Score = percentage of PD-L1 expressing tumor and infiltrating immune cells relative to the total number of tumor cells

## Post-platinum mUC population

IMvigor 210 (Cohort 2) and KEYNOTE-045 explored the use of atezolizumab and pembrolizumab, respectively, in the post-platinum mUC population. IMvigor 210 enrolled patients with locally advanced or mUC refractory to cisplatin-based chemotherapy. The SP142 rabbit antibody IHC assay was used to assess PD-L1 status in archival specimens as discussed above; PD-L1 positivity was defined as ≥10% PD-L1 positive immune cells in the tumor microenvironment (defined as 3+ in the phase I study of atezolizumab). While the objective response rate (ORR) of the entire cohort was 15%, the ORR was 26% (26/100) in PD-L1 positive patients, compared with only 9% (19/210) in PD-L1 negative patients (Table 1). These results seemed to confirm earlier studies showing the potential for PD-L1 as a predictive marker in mUC. Based on these results the Phase III IMvigor 211 trial randomized patients to atezolizumab or chemotherapy (paclitaxel, docetaxel or vinflunine) [7] with a primary endpoint of overall survival (OS) in PD-L1 positive subjects. The secondary endpoint of OS in the intention-to-treat (ITT) population was analyzed after the initial subset of PD-L1 positive cohort. While the ORR for the PD-L1 enriched cohort was 23% compared with 13% in the ITT cohort and confirmed prior findings, somewhat surprisingly, for the high PD-L1cohort there was no statistical difference in mOS when comparing atezolizumab to single agent chemotherapy (HR: 0.87; OS: 11.1 vs 10.6 months; *p* = 0.41) [7]. Interestingly, a significant difference in OS was observed in the ITT analysis for all patients treated with atezolizumab vs chemotherapy (HR: 0.85; OS: 8.6 vs 8.0 m; *p* = 0.038) [7]. It is still unclear why the ITT analysis for mOS was statistically significant, whereas the PD-L1 positive subset was not. One hypothesis is that the PD-L1 positive cohort was a smaller sample size and insufficiently powered to address the benefit in mOS for this cohort. Alternatively, the use of archival specimens may have confounded the true assessment of which patients were PD-L1-high at the time of study entry. Given these somewhat contradictory results, the accelerated FDA-approval for atezolizumab in mUC did not change after the results of IMvigor 2011, and further investigation of atezolizumab is underway in a Phase III study of platinum-naïve mUC patients. Based on these findings, it may be premature to select patients for therapy in clinical trials of CPIs based on the Ventana SP142 assay.

PD-L1 status did not predict for response in KEYNOTE-045 [1], a phase III trial which randomized 542 patients with mUC to treatment with either pembrolizumab or standard of care chemotherapy. KEYNOTE-045 utilized the 22C3 mouse antibody IHC assay (Dako/Agilent, CA, USA) using a combined positive score (CPS) to define PD-L1 positivity. The CPS was calculated as the percentage of PD-L1 expressing tumor and infiltrating immune cells relative to the total number of tumor cells; CPS ≥10% was considered PD-L1 positive. The ORR for all patients was 22%, and there was no difference between patients with CPS ≥ 10% compared with patients with a CPS < 10%. Thus, CPS was not predictive of response to treatment with pembrolizumab in this patient population. This result was in direct contrast to the data in non-small cell lung cancer (NSCLC), where CPS ≥ 50% does correlate with response to pembrolizumab [8]. More importantly, PD-L1 appeared to be a marker of poor prognosis, with patients who were PD-L1+ having poorer outcomes compared to patients who were PD-L1 negative by the CPS score, regardless of whether they received chemotherapy or pembrolizumab.

While patients with a positive Ventana SP142 PD-L1 test were almost three times more likely to respond to atezolizumab than those who were PD-L1 negative in IMvigor 210, the absolute difference in ORR between PD-L1 positive versus PD-L1 negative patients was not as large as seen in the phase 1 atezolizumab study. Furthermore, Dako 22C3 PD-L1 expression did not appear to be associated with any treatment response for pembrolizumab in KEYNOTE-045. Assuming that atezolizumab and pembrolizumab have similar clinical activity, it is worth exploring why, PD-L1 “positive” patients are not the same, and why these biomarkers may not have sufficient discriminatory power to predict response to a CPI. As discussed above, these IHC assays used completely different antibodies, and moreover, the assay characteristics define PD-L1 positivity based on staining different cellular populations (tumor cells versus immune cells versus tumor and immune cells). Yet another discrepancy includes the percentage of positive cells for the threshold of positivity within the assay.

Three additional CPIs have now been studied in and received accelerated FDA approval for the platinum-refractory population of mUC – nivolumab, durvalumab, and avelumab [5, 9, 10]. In the CheckMate 275 phase II trial of nivolumab, PD-L1 status was determined using the 28–8 rabbit antibody (Dako/Agilent, CA, USA). PD-L1 positive patients had a ORR of 28% (23/81) compared with an ORR of 16% (29/184) for PD-L1 negative patients [5]. For durvalumab and avelumab, the ability to predict the responders was somewhat greater, but in smaller, earlier phase studies. For durvalumab, a positive PD-L1 status (using the SP263 rabbit antibody, Ventana, AZ, USA) was defined as ≥25% of tumor cells or tumor infiltrating immune cells expressing PD-L1. Patients who met this definition had an ORR of 18% (27/98), compared with 5% (4/79) of patients who had a negative PD-L1 [4]. For avelumab, a positive PD-L1 status was defined as >5% PD-L1 expression by 73–10 rabbit antibody IHC (Dako/Agilent, CA, USA) on tumor cells [10]. The ORR was 54% (7/13) in the PD-L1 positive group, compared with 4% (1/24) in the PD-L1 negative group. While the use of PD-L1 as a predictive biomarker looks promising in these two studies, these were small studies, using different PD-L1 assays with different thresholds defining PD-L1 positivity. The difference in the prevalence of PD-L1 positivity across the trials suggests that different populations of “PD-L1 positive” patients are being captured by the different assays, which is further complicated by the different thresholds for positivity.

For example, in the durvalumab trial, 51% of patients were defined as PD-L1 positive, compared with only 16% of patients in the avelumab trial [4, 10]. This underscores the complexity of attempting cross-trial comparisons. In addition to different PD-L1 criteria, this may also be due to different inclusion criteria – the avelumab trial required at least one previous line of treatment but the durvalumab trial did not. As clinical development moves forward for each of these agents, further assay standardization, test characteristics, definitions of the “biomarker-positive” population all need to be addressed.

## Platinum ineligible mUC

Due to a variety of factors, including renal or hearing impairment, poor performance status, and neuropathies, 30% to 50% of patients with chemotherapy-naïve advanced UC are not candidates for platinum-based chemotherapy [11]. Cohort 1 of IMvigor 210 and KEYNOTE-052 explored the use of atezolizumab and pembrolizumab, respectively, in platinum-ineligible patients with mUC. Using the SP142 Ventana assay, PD-L1 positive patients in IMvigor 210 had an ORR of 28% (9/32) compared to 20% (18/87) in those who were PD-L1 negative [12]. Thus, the difference in ORR between PD-L1 positive and PD-L1 negative patients was minimal. In KEYNOTE-052, PD-L1 (with the 22C3 assay) appeared to be associated with higher response rates: 51% (41/80) of patients with a CPS ≥ 10% had an objective response compared with 23% (42/185) of patients with a CPS < 10% [13]. It should be emphasized that these studies in chemotherapy-naïve, cisplatin ineligible patients were both single-arm phase II studies, and assessment of the predictive capacity of PD-L1 would be better explored in appropriately powered Phase III studies. The disparate results again suggest that a single PD-L1 score is not sufficient to predict the population of patients who will respond to immune CPIs. Multiple Phase III studies are underway (JAVELIN bladder 100 with avelumab [NCT02603432], IMvigor 130 with atezolizumab [NCT02807636], and DANUBE with durvalumab and tremelimumab [NCT02516241]) and will further explore the predictive and prognostic capacity of PD-L1.

Based on the data presented above, in which patients with mUC may respond to PD-1/PD-L1 blockade even if their archival tumor lacks PD-L1 expression, we do not recommend PD-L1 clinical testing in UC patients. While in some studies PD-L1 positivity may identify patients more likely to have an objective response, and combined tumor/microenvironment testing may further enrich for responders, in others studies this biomarker has no discriminatory power, and given the conflicting results treatment with a CPI should not be withheld based on PD-L1 status in mUC. Prospective studies of PD-L1 as a predictive biomarker are needed, with consideration for contemporary/recent biopsies, tumor heterogeneity assessments, and expression in tumor vs normal immune cells.

## Renal Cell Carcinoma (RCC)

Nivolumab received FDA approval for the treatment of patients with advanced RCC refractory to first-line vascular endothelial growth factor (VEGF) inhibitors, demonstrating improved OS compared to everolimus (median OS 25 vs 19.6 months, HR 0.73, *p* = 0.002) [14]. Tumor PD-L1 membrane expression was assessed using the 28–8 Dako assay, and positivity was defined with 2 separate cut-offs as either ≥1% or ≥5% of tumor cells (Table 2). PD-L1 expression was prognostic of survival – those with higher PD-L1 expression had poorer survival than those with lower PD-L1 expression. Median OS was 21.8 months for patients with ≥1% PD-L1 expression compared with 27.4 months for patients <1% PD-L1 expression in each nivolumab-treated cohort [14]. Nivolumab improved median OS in all patients compared to everolimus, regardless of PD-L1 status [15]. Therefore, PD-L1 was not a reliable predictive biomarker of treatment response. An interesting observation, however, is that many poor risk and sarcomatoid tumors have high levels of PD-L1 expression in their archival tumors, and this subset of patients actually had the greatest relative benefit with nivolumab over everolimus [16, 17]. These data suggest that aggressive clear cell RCC tumors upregulate PD-L1 and may be more vulnerable to checkpoint blockade.

**Table 2:** Summary of assays and response rates in immune checkpoint inhibitor trials in metastatic renal cell carcinoma

Ref	CPI	Drug	Target	Antibody for PD-L1 IHC Assay	Definition of PD-L1 positivity	ORR (PD-L1+)	ORR (PD-L1-)	ORR All Patients
[14]	Single Agent	Nivolumab	PD-1	Rabbit 28–8 (Dako)	PD-L1 ≥ 5% (TC)	45% (13/29)	18% (14/78)	25% (27/107)
[18]	Single Agent	Atezolizumab	PD-L1	Rabbit SP142 (Ventana)	IHC 1/2/3 IC^a^	18% (6/33)	9% (2/22)	15% (8/55)
[25]	Combination^b^	Nivolumab and Ipilimumab	PD-1 and CTLA-4	Rabbit 28–8 (Dako)	PD-L1 ≥ 1% (TC)	58% (58/100)	37% (105/284)	42% (163/384)

*TC* tumor cells, *IC* percentage of PD-L1 positive immune cells in the tumor microenvironment, *ORR* overall response rate

^a^IHC 1 is ≥1%, IHC 2 is ≥5%, IHC 3 is ≥10%

^b^IMDC Intermediate/poor risk

Atezolizumab has also been investigated in mRCC. The expansion cohort of a phase Ia trial enrolled 70 patients with treatment refractory mRCC; all patients were treated with atezolizumab [18]. Enrollment started with all patients regardless of PD-L1 status, but was later limited to tumors which expressed PD-L1 IC2 or IC3 (≥5% IC positive for PD-L1) by the SP142 Ventana assay. The number of patients in the trial was small but those defined as having increased PD-L1 expression had a higher ORR than those lacking PD-L1 expression (18% vs 9%, Table 2).

Atezolizumab has also been investigated in the frontline setting in combination with bevacizumab, a VEGF inhibitor [19]. Bevacizumab had demonstrated efficacy previously with immunotherapy, in combination with interferon alpha-2a (IFNa) among a population of untreated mRCC. The combination improved PFS in two major clinical trials, AVOREN and CALGB 90206 [20, 21]. IMmotion 150 was a phase II trial for untreated mRCC in which patients were randomized to atezolizumab in combination with bevacizumab, atezolizumab alone, or sunitinib. Patients were allowed to crossover to the combination arm after disease progression on either atezolizumab or sunitinib. PD-L1 expression was measured based on the Ventana SP142 IHC assay, and patients with a PD-L1 expression ≥1% were considered PD-L1 positive. The ORR in the combination arm among PD-L1 positive patients was 46% compared to 28% in the atezolizumab arm alone, and 27% in the sunitinib arm. The hazard ratios for the combination arm compared with sunitinib were 0.64 (95%CI 0.38–1.08, *p* = 0.095) and 1.03 (95%CI 0.63–1.67, *p* = 0.917) for the atezolizumab alone vs sunitinib arm. Early phase trials combining immune checkpoint inhibitors with small molecule VEGF receptor inhibitors inpatients enrolled regardless of PD-L1 status have shown promising results with ORRs ranging from 67% to 100%. These studies demonstrate a signal for potentially improved overall response rates for patients treated with combination therapy. Several phase III studies are currently underway investigating checkpoint inhibitors in combination with VEGF-targeted therapy for patients with mRCC [22–24].

Immunotherapy CPI combinations have proven effective in melanoma, and CheckMate-214 was the first in mRCC to use combination of CTLA-4 and PD-1 inhibitors. CHECKMATE 214 was a phase III trial which randomized 1040 patients with metastatic clear cell RCC to treatment with either combination nivolumab-ipilimumab or sunitinib. Co-primary endpoints included ORR, progression free survival (PFS), and OS, specifically in the IMDC intermediate or high risk population. Secondary endpoints included PFS and OS for the intention to treat population (including favorable risk). Nivolumab-ipilimumab improved both median OS (not reached (NR) vs 26.0 months, HR 0.63, *p* < 0.0001) and ORR (42% vs. 27%, p < 0.0001) in patients with intermediate-high risk disease [25]. PD-L1 positivity was defined using the 28–8 assay (Dako/Agilent, CA, USA) as >1% of tumor cells. In the IMDC intermediate or high risk patients, ORR was 37% in PD-L1 negative patients 58% and PD-L1 positive patients (Table 2) [25]. In the PD-L1 negative patients with IMDC intermediate or high risk, PFS did not differ between those treated with nivolumab-ipilimumab versus sunitinib (HR 1.00, *p* = 0.98), whereas in PD-L1 positive population, there was a large difference in PFS between these two groups (HR 0.48, *p* = 0.0003) [25]. However, both PD-L1 positive and PD-L1 negative patients benefited with improved overall survival. Therefore, PFS was not a good surrogate endpoint for survival benefit in the PD-L1 negative cohort. Given the small difference in response rates in PD-L1 positive versus PD-L1 negative patients, as well as the improvement in mOS for these patients, the role for PD-L1 testing remains unclear – negative PD-L1 status would not necessarily select patients who would not benefit from nivolumab-ipilimumab. Indeed, in the IMDC favorable risk group, ORR favored sunitinib over nivolumab-ipilimumab (52% vs. 29%, *p* = 0.0002) [25]. Further data needs to be presented regarding PD-L1 status in patients with favorable risk disease, and their survival analyses. For now, however, PD-L1 status is not clinically useful in informing treatment decisions in mRCC.

Further correlative work has also emphasized the ineffectiveness of PD-L1 as a predictive biomarker in mRCC [26]. Primary tumor and metastatic tumors have discordant PD-L1 expression – in one pathology-based study, discordant PD-L1 expression was detected in 21% (11/53) of cases, suggesting that analysis of metastatic biopsies may be necessary to form an accurate assessment of PD-L1 expression [27]. Moreover, PD-L1 expression is dynamic and can arise after treatment as a form of treatment resistance [28]. This further emphasizes the inadequacy of archival tissue to assess such a dynamic biomarker. Based on the data above, at this time, we do not recommend PD-L1 clinical testing in metastatic RCC give the lack of clear predictive value in any clinical setting.

## PD-L1 as a biomarker

The clinical trials for mUC and mRCC mentioned above highlight the numerous challenges facing PD-L1 as a biomarker. First, there is no standardized format to assess PD-L1 immunohistochemically. Several pharmaceutical companies have established partnerships with diagnostic companies to design companion PD-L1 assays. These assays are in a sense more different than they are similar. The only similarity between the assays is that they are all IHC tissue-based assays to detect membrane expression of PD-L1. Critical differences include which components are included for the purposes of scoring (tumor cells vs. immune cells), as well as staining thresholds to define PD-L1 positivity [29]. Thus, individual patients may have discordant results depending on the assay. This is evident when examining the data supporting the use of durvalumab for post-platinum UC patients. When the authors defined PD-L1 positivity by only one component, immune cells (IC) or tumor cells (TC), there did not appear to be a clean separation between the responders and non-responders [9]. However, when PD-L1 status was defined by both components (≥25% expression in either IC or TC), a clear demarcation between responders and non-responders emerged. This suggests that perhaps a better predictive marker may be incorporating both PD-L1 status from both IC and TC, rather than using one by itself.

Second, because these clinical trials have used different companion diagnostic assays, we are unable to indirectly compare the effectiveness of one drug in a “PD-L1 positive” population compared to another drug in a “PD-L1 positive” population. The first two concerns have been ameliorated in the diagnostic testing for non-small cell lung cancer (NSCLC). Ratcliffe et al. studied the concordance between three commercially available PD-L1 IHC assays for NSCLC patients including Ventana SP263 (durvalumab), Dako 22C3 (pembrolizumab), and Dako 28–8 (nivolumab) and found >90% concordance at several levels (1%, 10%, 25%, 50%) of PD-L1 expression [30]. The separate Blueprint PD-L1 study also studied the 4 different PD-L1 antibody assays in NSCLC, scored independently by three expert pathologists. However, the authors showed that PD-L1 status would have been classified differently depending on PD-L1 assay in 37% (14 of 38) of the cases [31]. This data suggest that these assays may be used to aid in the therapeutic decision making for a NSCLC population, but they may have variable concordance in defining PD-L1 positivity. Similar studies to compare the PD-L1 assays have not been performed in advanced UC or RCC.

Lastly, there are both biological and technical challenges which further complicate PD-L1 testing. This has been well reviewed in the literature [32, 33]. Biological challenges include intratumoral and intertumoral heterogeneity of PD-L1 expression, a temporal evolution of PD-L1 status particularly during the development of treatment resistance, and variation in PD-L1 expression according to the level of tissue hypoxia [27]. PD-L1 is particularly dynamic and the use of archival specimens for assigning PD-L1 status severely limits the classification of patients at the time of treatment. PD-L1 status in the archival specimen may not reflect the true PD-L1 status of a patient when started on CPI treatment. Technical challenges include variation in PD-L1 expression due to time in formalin, variation in the affinity of the anti-PD-L1 antibody, and standardization of the systems for amplification and discordance between assay definitions on what constitutes a positive PD-L1 signal.

## Biomarkers beyond PD-L1

There are several other predictive biomarkers under examination for mUC and mRCC, including tumor mutational burden (TMB), mismatch repair status, gene expression profiles (GEP), TCGA (The Cancer Genome Atlas) profiling, tumor infiltration lymphocytes (TILs), and PD-L2.

## Tumor mutational burden and MSI status

It has been observed that tumors with a higher mutational burden may have a better response to immunotherapy [34]. Higher tumor mutational burden (TMB) is theoretically associated with increasing neo-antigens that may be better recognized by the immune system. A high prevalence of insertions/deletions (indels) may also be predictive of IC response due to associations with neoantigen load in RCC, suggesting that the specific types of genomic alterations in tumors may be critically important for antigen presentation and recognition [35].

In mUC, data from IMvigor 210 further suggest that high TMB may not be only prognostic of survival, but potentially predictive of response [3, 13]. Patients in the highest quartile of TMB had a significantly longer mOS when treated with atezolizumab compared with those in quartiles 1–3 (*p* = 0.0041) [13]. TMB did not correlate with TCGA subtype, immune cell subgroups, or smoking history. High TMB patients have also been shown in Checkmate 275 to have a better ORR (31.9% vs 17.4%, *p* = 0.002) and median PFS (3.02 months vs. 1.87 months) [36]. Recently, high TMB was also found in mUC patients who also harbored *ERBB2* (HER2) and *ERBB3* (HER3) mutations [37]. These data suggest targets for rational combinations of mUC-targeting treatments. These data suggest that TMB can potentially predict for treatment responses to immune CPIs, but more prospective studies are needed to elucidate its true predictive role.

Not surprisingly, patients with the highest mutational burden often harbor specific DNA damage response defects, such as microsatellite instability (MSI-H) or are mismatch repair deficient (dMMR) [38]. Thus, clinical trials enriching for this population have found that patients with mismatch repair defects have some of the highest response rates to PD-1 blockade [39]. Based on the cumulative data from five clinical trials totaling 149 patients, the FDA has approved pembrolizumab for tumors that have been identified as MSI-H or dMMR [40]. This tumor agnostic approval of pembrolizumab was the first of its kind. Based on data from five KEYNOTE trials, pembrolizumab had an overall response rate of 40% amongst the population who were identified as MSI-H and dMMR, and it is worth noting that 78% of the patients who responded had a response rate of 6 months or longer [39, 41]. MSI-H a major feature of patients with HNPCC or Lynch syndrome (Hereditary Nonpolyposis Colorectal Cancer). While colon and endometrial cancer are the two most common HNPCC associated cancers, upper tract urothelial carcinomas (UT-UC) rank third, with a prevalence of 5% [42, 43]. For patients with UT-UC, further history should be obtained to elucidate their risk factors for Lynch syndrome, and if appropriate, proceed with genetic counseling and testing.

## Gene expression profiles (GEP)

Gene expression profiling has demonstrated utility as a predictive biomarker to chemotherapy and immunotherapy [44–47]. The expression of interferon gamma is one of the key biomarkers that has been explored as a predictive biomarker for these agents. When abnormal cellular growth occurs as a result of exposure to radiation, smoking, or chronic viral infections, the innate and adaptive antitumor response is initiated which leads to the production of interferon gamma. Interferon gamma is produced by activated T cells, natural killer cells (NK) cells, the tumor microenvironment, and can lead to upregulation of both PD-L1 and PD-L2. Through a feedback loop, interferon gamma can also upregulate the expression of immunosuppressive molecules such as IDO1. Thus, interferon gamma is key in protecting the host from developing tumors, but it also facilitates tumor escape mechanisms from the immune system [48].

Ayers et al. evaluated 680 tumor and immune related genes using a NanoString nCounter platform and found that the genes most able to separate responders from non-responders of PD-1 therapy were genes linked to interferon gamma signaling [49]. The initial panel of genes was developed based on a small cohort of melanoma patients but was further expanded using a data set spanning nine cancer types, which ultimately resulted in an 18 GEP which could predict response to pembrolizumab across multiple solid tumors. This GEP was used in the trial examining pembrolizumab in the first line platinum ineligible UC population [50]. The authors found that the GEP score was significantly associated with a treatment response (*p* < 0.0001). GEP score could correctly predict 70 of the 81 responders, compared to the PD-L1 CPS cutoff, which predicted only 41 of the 81 responders. While this is hypothesis generating, the utility of GEP still needs to be validated in large prospective clinical trials.

## Intrinsic molecular subtypes

Consensus clustering based on gene expression data from TCGA has distinguished two major intrinsic subtypes of high grade bladder cancer – luminal and basal [51]. Rosenberg et al. explored the link between these unique molecular subtypes of bladder cancer and their response to PD-L1 inhibition [3]. Basal subtype tumors had increased PD-L1 tumor cell expression (39% in basal vs 4% in luminal, *p* < 0.0001) and increased PD-L1 immune cell prevalence (60% vs 23%, p < 0.0001). However, the increase of PD-L1 expression in the basal subtype did not correlate with an objective response. Response to atezolizumab in this post-platinum population was much higher in the luminal cluster II subtype compared with all other subtypes. This relationship was again observed in the front line platinum-ineligible population with the luminal II samples having a higher response rate compared to the other molecular subtypes – however, the sample size was not sufficient to reach statistical significance [12]. Further studies are necessary to confirm if luminal cluster II may be a predictive biomarker of response.

## PD-L2

PD-L2 is another ligand capable of binding to PD-1. While its structure is quite similar to PD-L1, the exact function of PD-L2 is controversial [52]. PD-L2 plays a role in regulating T cell immune response and immune tolerance - some studies have shown that PD-L2 is an inhibitory co-stimulatory molecule whereas others have shown that it is a positive costimulatory molecular that functions through a different receptor than PD-1 [53, 54]. What is known, however, is that the expression pattern of PD-L1 and PD-L2 is distinct, that both PD-L1 and PD-L2 compete to bind to PD-1, and that PD-L2 may be capable of binding to other receptors in addition to PD-1 [55].

In RCC, while PD-L1 expression was observed in only 9% of cases in a series of 425 patients, PD-L2 expression was observed in 50% of cases [56]. PD-L1 and PD-L2 were evaluated retrospectively amongst a cohort of 20 selected patients – 8 patients who had a durable clinical benefit (DCB) for more than 12 months and 12 patients with limited clinical benefit (LCB), defined as less than 6 months of benefit [26]. As a predictive biomarker, when PD-L1 or PD-L2 were analyzed separately, there did not appear to be an association with clinical benefit – however, when analyzed together, a significant association was found when using a PD-L1 cutoff score of 1% and PD-L2 cutoff score of 5%. 88% (7/8) of the DCB cohort met this metric of being positive for either PD-L1 or PD-L2, or both, compared to 36% (4/11) of the LCB group for the same metric [26]. The ability for PD-L2 to predict response has also been demonstrated in NSCLC [57]. Larger studies are necessary to validate this relationship.

## Tumor infiltrating cytotoxic T lymphocytes (TILs)

CPIs are known to enhance the activity of the adaptive immune system by recruiting CD8 positive cytolytic T cells into the tumor microenvironment [58]. CD8 positive T cell density and CD8 positive density at the invasive margin, as well as clonality within the T-cell repertoire have been previously described as predictive biomarkers for response to CPI therapy in metastatic melanoma [58]. A special population of CD8 positive T cells have been identified as “partially exhausted” cytotoxic T lymphocytes (peCTL), which are tumor-infiltrating CD8+ T cells which express high levels of cytotoxic T lymphocyte–associated antigen 4 (CTLA-4) [59]. peCTLs have been shown to strongly correlate with response to anti-PD1 therapy in metastatic melanoma [59, 60]. Interestingly, while high levels of peCTLs are associated with a response in anti-PD1 therapy, a lower peCTL level was correlated with a higher ORR with combination immunotherapy. This suggests that peCTL levels may be useful when deciding between single or combination immunotherapy in the future.

High T cell infiltration and clonality in advanced UC has also correlated with treatment response [58]. Increased TILs within the tumor microenvironment, particularly in UC, have been found to correlate with improved disease free survival and overall survival [61]. In a retrospective evaluation of 31 patients with muscle invasive UC, patients with more than 8 CD8 TILs had a median survival of more than 80 months, compared to a median survival of 13 months for those with less than 8 CD8 cells [61]. In IMvigor 210 of atezolizumab in platinum-refractory mUC, the inflammatory CD8 cytotoxic T-cells within the tumor microenvironment was associated with objective response to atezolizumab (*p* = 0.0265) [3]. These tests are more cumbersome but may ultimately prove to be more useful as biomarkers for response to CPI therapy.

## Composite immune biomarkers

Ultimately, a single biomarker may not adequately predict response to PD-1 immune checkpoint therapies, and a combination of factors may need to be taken into account to predict responses. Based on the level of CD8A and PD-L1 expression, Ock et al. described four distinct tumor microenvironment immune types (TMIT) [62]. Tumors with an elevated CD8A and PD-L1 expression were grouped as Type I TMIT. Type I TMIT tumors had a significantly higher mutational burden compared with the other types of tumors, higher number of neoantigens, and were also associated with PD-L1 amplification, all characteristics which imply responsiveness to PD-1 and PD-L1 inhibition. This suggests that TMIT may be a more comprehensive score as a predictive biomarker for these CPIs. Prospective clinical trials using the TMIT framework are necessary. These types of composite assessments which combine multiple biomarkers within the same platform are currently in development. A concern about using composite biomarker panels, however, is both their clinical utility (will it be feasible to wait for multiple assays to return before starting CPI therapy?) as well as increased costs associated with multi-omic and IHC profiling.

## Gastrointestinal microbiome

It is well established that the gut microbiota plays a critical role in both innate and adaptive immune homeostasis [63]. Thus, it would be reasonable to suspect that the fecal microbiome may contain potential predictors of CPI as well as predictors of CPI induced colitis. Both of these questions have been investigated. In germ-free or antibiotic treated mouse models, CTLA-4 blockade was ineffective at preventing tumor progression [64]. However, after re-colonization of germ free or antibiotic treated mice with several bacterial species including *B. fragilis*, the mice were able to recover an anticancer response to CTLA-4 blockade. This finding is similar to that reported by Sivan et al., which found that the best responses to PD-L1 blockade required specific Bacteroides - mice which were given oral Bifidobacterium improved tumor control to the same degree as those given an anti-PD-L1 agent [65]. With regards to predicting tumor toxicity, Dubin et al. have discovered that the presence of specific microbiota phylotypes can help predict a patient’s risk of developing colitis following CTLA-4 blockade [66]. These studies suggest that the further research on the gastrointestinal microbiome may yield biomarkers for predicting response and provide strategies to maximize clinical benefit.

## Conclusion

The FDA approval of several checkpoint inhibitors for the treatment of mUC and mRCC represents a major landmark for patients and oncologists. However, it is critical that we choose the right patients, given that these drugs have the potential for not only physical toxicity but also financial toxicity, with costs of up to $20,000 per month [67]. Selecting patients who will respond to PD-1 and PD-L1 blockade remains a challenge. Data from the clinical trials which led to the approvals of PD-1 and PD-L1 directed therapies in mUC and mRCC show that the use of PD-L1 expression alone is insufficient and inefficient at predicting treatment response. The future of predictive biomarkers likely involves a combination of many biologic variables, some of which have been mentioned above. Of these, tumor mutational burden shows promise in predicting for treatment responses to immune CPIs. Ultimately, a single biomarker may not be enough to determine treatment response, and comprehensive biomarkers incorporating several or all of these biomarkers are currently in development as predictive biomarkers. These composite biomarkers ultimately will need to be prospectively validated in the context of therapeutic clinical trials.
